# Supplementary material for: Characterization of Bacillus velezensis EV17 and K-3618 and Their Polyketide Antibiotic Oxydifficidin, an Inhibitor of Prokaryotic Translation with Low Cytotoxicity
Source: Int J Mol Sci. 2025 Dec 5;26(24):11777. doi: 10.3390/ijms262411777 (PMC12732368; doi:10.3390/ijms262411777)
Supplement: Supplementary file 1 [file ijms-26-11777-s001.zip › ijms-3893330-supplementary.pdf]

Supplementary material

This file contains:

S1. Detailed Synthesis of Fluorescent Thiostrepton Derivative (Ths-FITC) 2
Chemicals and Materials 2
Chromatography and Mass Spectrometry 2
Synthetic Procedures 2
Figure S1. 3
Figure S2. 4
Figure S3. 5
Figure S4. 6
Figure S5. 7
Figure S6. 8
Figure S7. 9
Figure S8. 10
Figure S9. 11
Table S1. 12
Table S2. 13

S1. Detailed Synthesis of Fluorescent Thiostrepton Derivative (Ths-FITC)

Chemicals and Materials

The following reagents and solvents were used: amino acids, their derivatives, and 2-chlorotrityl chloride resin for solid-phase peptide synthesis (2CTC Resin) (Iris Biotech, Marktredwitz, Germany); HBTU (hexafluorophosphate benzotriazole tetramethyl uronium), DIPEA (N,N-diisopropylethylamine), thiostrepton (Ths) (Sigma-Aldrich, Steinheim, Germany); CH_2Cl_2, dimethylformamide (DMF), trifluoroacetic acid (TFA), triethylamine (TEA) (PanReac AppliChem, Darmstadt, Germany); chloroform, diethylamine (Chimmed, Moscow, Russia); acetonitrile (Biosolve Chimie, Dieuze, France); methanol, piperidine (Merck, Darmstadt, Germany); fluorescein-5-isothiocyanate (FITC), 2,2,2-trifluoroethanol (TFE) (Fluka AG, Buchs, Switzerland); triisopropylsilane (TIPS) (TCI, Tokyo, Japan); propionic anhydride (Chemical Line, St. Petersburg, Russia).

Chromatography and Mass Spectrometry

TLC on Kieselgel 60 F254 (Merck, Darmstadt, Germany) and column chromatography on silica gel 60 (0.063–0.200 mm, Macherey-Nagel, Dueren, Germany) were performed; UV-absorbing compounds were detected with a Camag UV cabinet (Camag, Muttenz, Switzerland).

Preparative HPLC was performed on a Knauer Smartline system (Knauer, Berlin, Germany) with a Beckman Coulter Ultrasphere ODS column (5 μm, 250 × 10 mm), using Smartline 1050 pumps, UV 2520 detector, and Smartline Manager 5050. ClarityChrom 8.1 software was used. Separation was performed at 5 mL/min and ambient temperature using an appropriate gradient of CH_3CN in 0.1% TFA.

Liquid chromatography–mass spectrometry (LC-MS) was performed using a UPLC/MS/MS system comprising an Acquity UPLC chromatograph and a TQD quadrupole mass spectrometer (Waters Corporation, Milford, MA, USA). Positive ions were detected by ESI-MS using an Acquity BEH C18 column (1.7 μm, 50 × 2.1 mm) at 0.5 mL/min and 35 °C, with elution via a 4-min gradient of 5–100% CH_3CN in 20 mM HCOOH.

MALDI-TOF analysis was performed with an UltrafleXtreme mass spectrometer (Bruker Daltonics, Bremen, Germany) using a UV Nd laser in reflectron positive-ion mode.

Synthetic Procedures

Preparation of truncated thiostrepton derivative (truncThs) [1]. Thiostrepton (100 mg, 0.06 mmol) was dissolved in $CHCl_3$ (5 mL) and diethylamine (0.5 mL) was added dropwise at 0 °C. After 5 min, the mixture was stirred at room temperature for 2.5 h. Volatiles were removed by co-evaporation with toluene, and the residue was purified by silica gel column chromatography (5% MeOH in $CHCl_3$) to afford truncated thiostrepton (truncThs) as colorless powder. Yield: 65 mg (68%); TLC: R_f ($CHCl_3$:CH_3OH, 9:1) 0.47; LC-MS: t_R = 2.56 min, *m/z* calculated for $[C_{69}H_{82}N_{18}O_{17}S_5+H]^+$ —1595.48; found—1595.03.

FITC-βAla-Ser-Gly-Ser-Gly-Cys-OH. Fluorescently labeled peptide was synthesized using the standard Fmoc solid-phase peptide synthesis protocol using 2-chlorotrityl chloride resin (150 mg, 0.7 mmol/g) and HBTU/DIPEA activation, employing 4 eq. of Fmoc-protected amino acid at each coupling step. Capping was performed using 10 eq. of propionic anhydride and 10 eq. of DIPEA in DMF. Fmoc deprotection was carried out using 20% piperidine in DMF. The fluorescent FITC label was attached to the peptide during SPPS using solution of 4 eq. (relative to the peptide loading) of FITC and 8 eq. of DIPEA in DMF. Cleavage from the resin was performed with TFA–TIPS–H_2O (95:2.5:2.5, *v*/*v*) at 10 mL/g of peptidyl polymer for 1 h at room temperature under an argon atmosphere. The final crude FITC-labeled peptide (8.2 mg, 9.4 μmol) was obtained from 23 mg of peptidyl polymer and used immediately in the next stage. LC-MS: t_R = 1.58 min, *m/z* calculated for $[C_{37}H_{39}N_7O_{14}S_2+H]^+$—870.21; found—869.04.

Fluoresceine-labeled truncated peptidyl-thiostrepton (Ths-FITC). The fluorescent peptide FITC-βAla-Ser-Gly-Ser-Gly-Cys-OH (8.2 mg, 9.4 μmol, 1.5 eq) was dissolved in TFE (1 mL), and NaPi buffer (0.5 mL, 50 mM, pH 8.8) was added. Then truncThs (10 mg, 6.3 μmol, 1 eq) and TEA (17 μL, 125 μmol, 20 eq) were added to the solution. The reaction was stirred under argon for 24 h, then concentrated and purified by preparative HPLC (linear gradient of 20–60% CH_3CN in 0.1% TFA during 20 min, t_R = 12.5 min) to yield Ths-FITC as a yellow

powder after lyophilization. Yield: 2.7 mg (17%); TLC: R*f* (1-buthanol:H_2O:CH_3COOH, 4:1:1) 0.44; LC-MS: t_R = 1.87 min, *m/z* calculated for $[C_{106}H_{121}N_{25}O_{31}S_7+2H]^{2+}/2$—1232.84; found—1232.08. MALDI-TOF MS: *m/z* calculated for $[C_{106}H_{121}N_{25}O_{31}S_7+H]^{+}$—2464.7; found—2464.7.

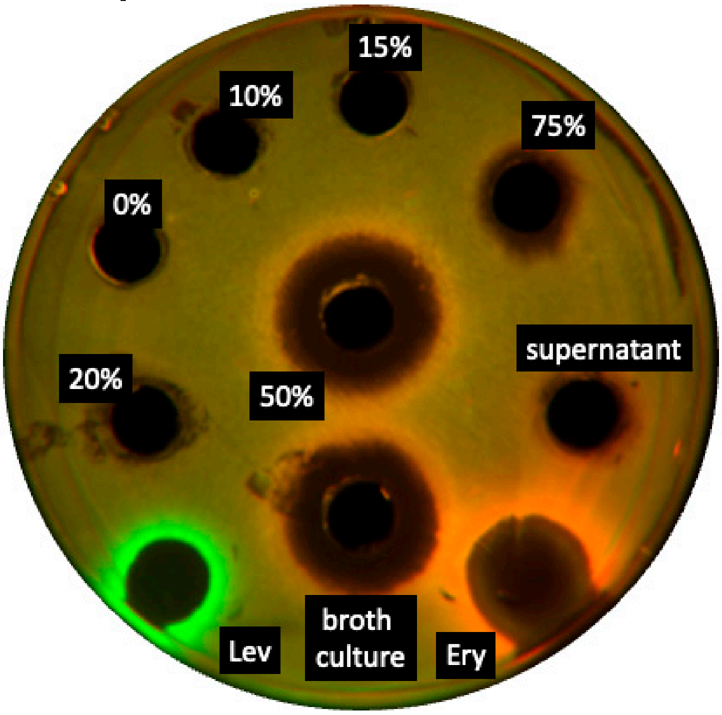

Figure S1.

Agar plates coated with *E. coli lptD*^{mut} pDualrep2.1 reporter strain and spotted with culture fluid of the producer *Bacillus* sp. EV17, grown on Org79 medium, and samples obtained during EV17 broth culture purification via solid-phase extraction on LPS-500-H sorbent, along with two antibiotic controls – erythromycin (Ery) (5 mg/ml) and levofloxacin (Lev) (25 mg/ml). Notations: culture broth of EV17 strain; 0, 10, 15, 25, 50 and 75% – elution with solutions of acetonitrile in water of the indicated volume concentration; supernatant – aqueous fraction collected after the broth culture was passed over the sorbent.

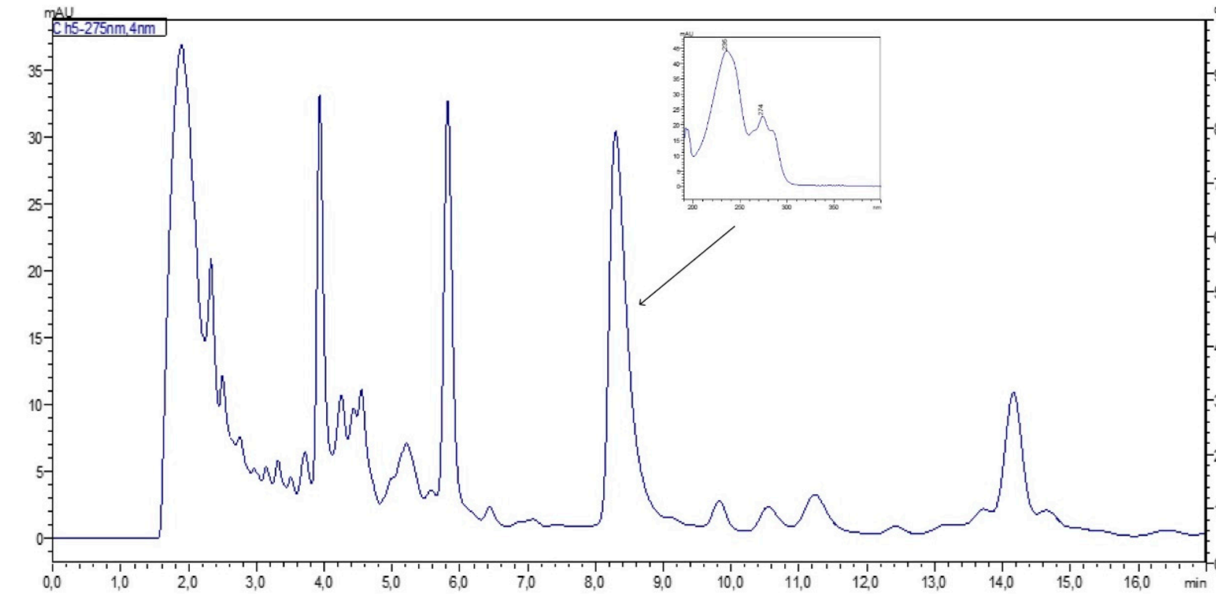

Figure S2.

HPLC of the active fraction (Gemini NX C18 150 x 10 mm, 5 µm, 110 Å; eluent solvent A - 10 mM NH_4OAc, pH 5, solvent B – MeCN; isocratic elution at 40% of solvent B; flow rate 3 mL/min, UV 275 nm. Active peak is at 8.3 min. UV spectra of the active peak is shown near the peak.

A

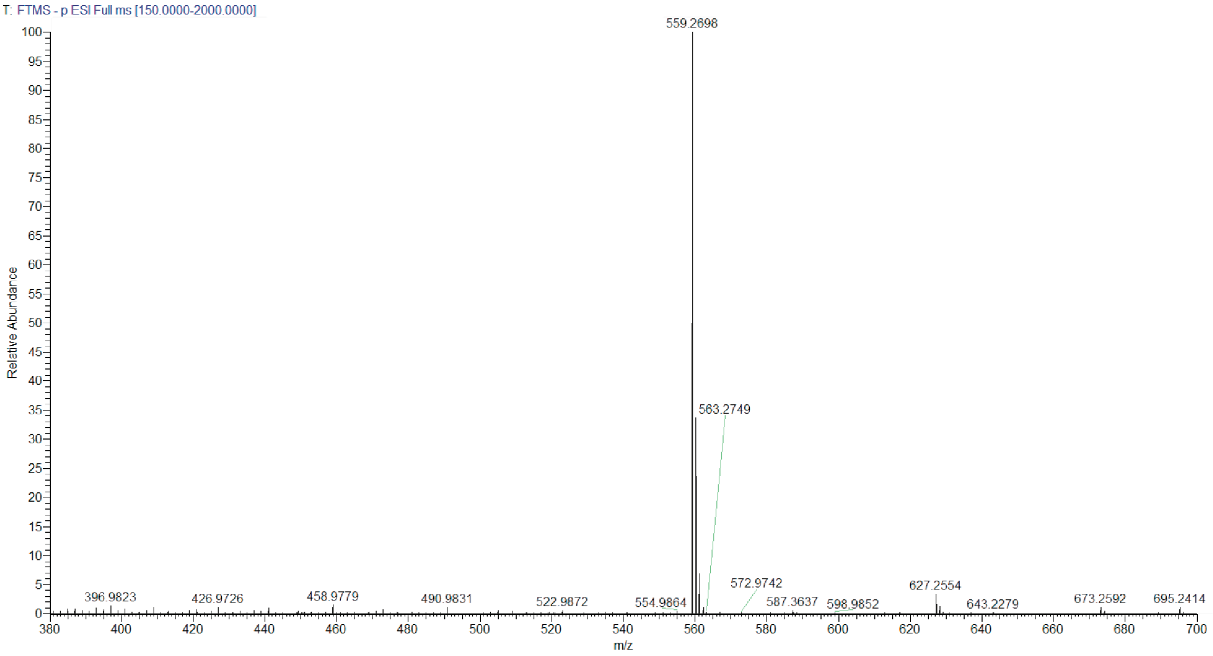

B

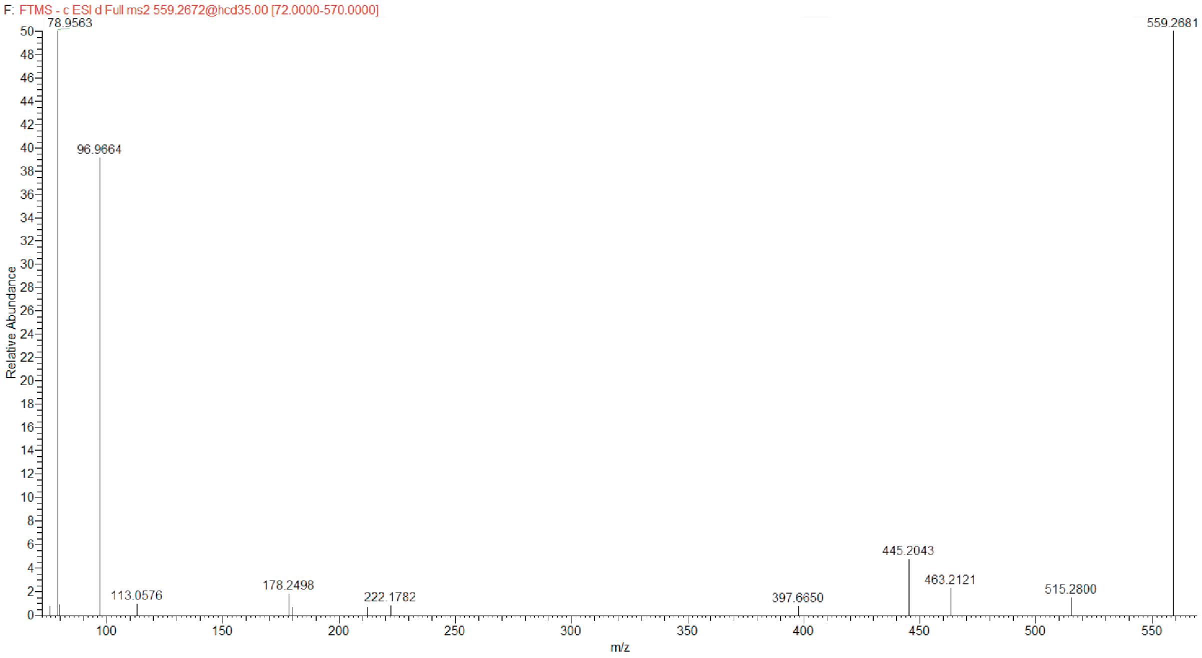

Figure S3.

(A) Negative-ion mode MS1 spectra of oxydifficidin; (B) HCD mass spectra of the parent ion [M-H]- at m/z 559.2672

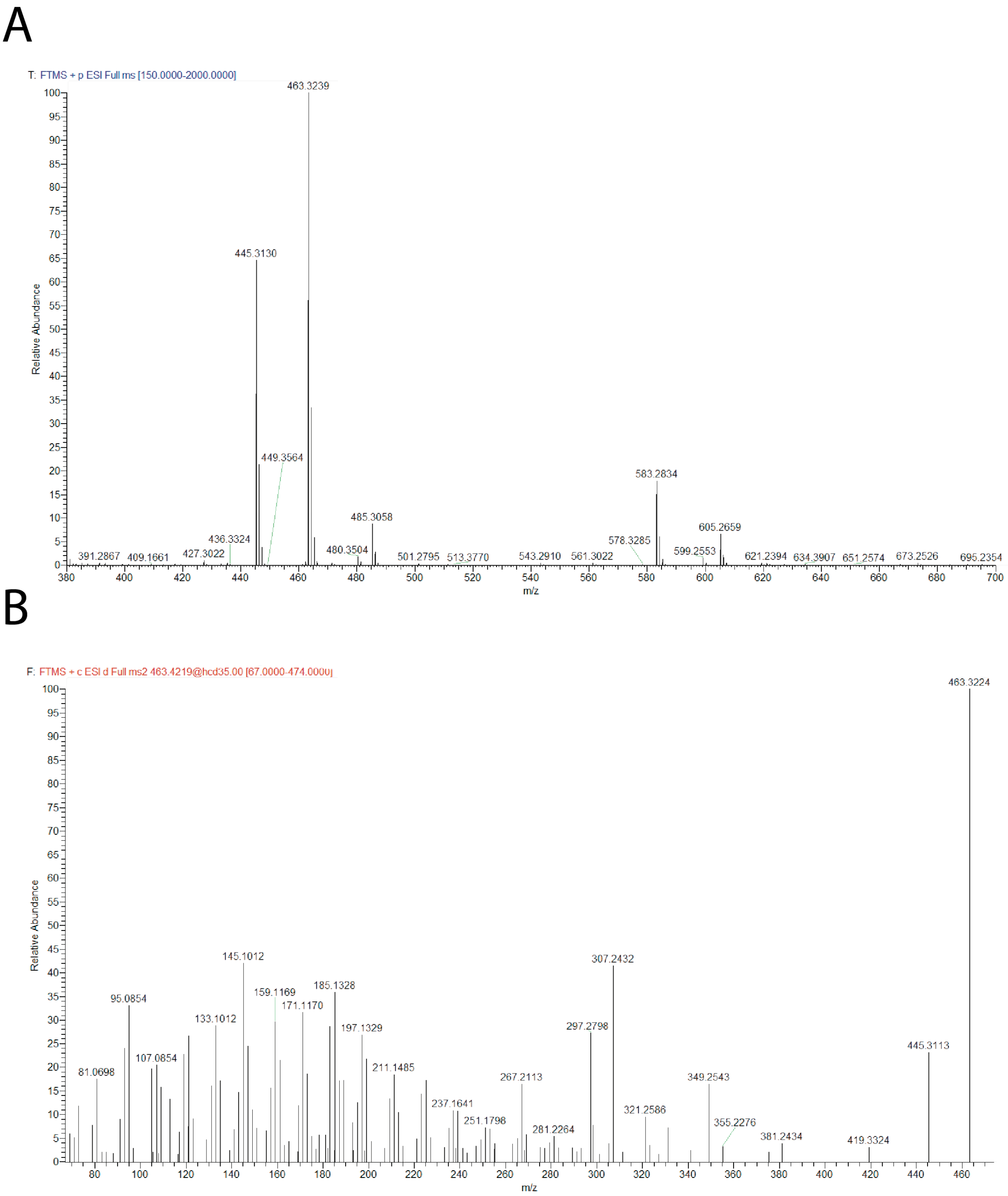

Figure S4.

(A) Positive-ion mode MS1 spectra of oxydifficidin; (B) HCD mass spectra of the precursor ion at m/z 463.4219

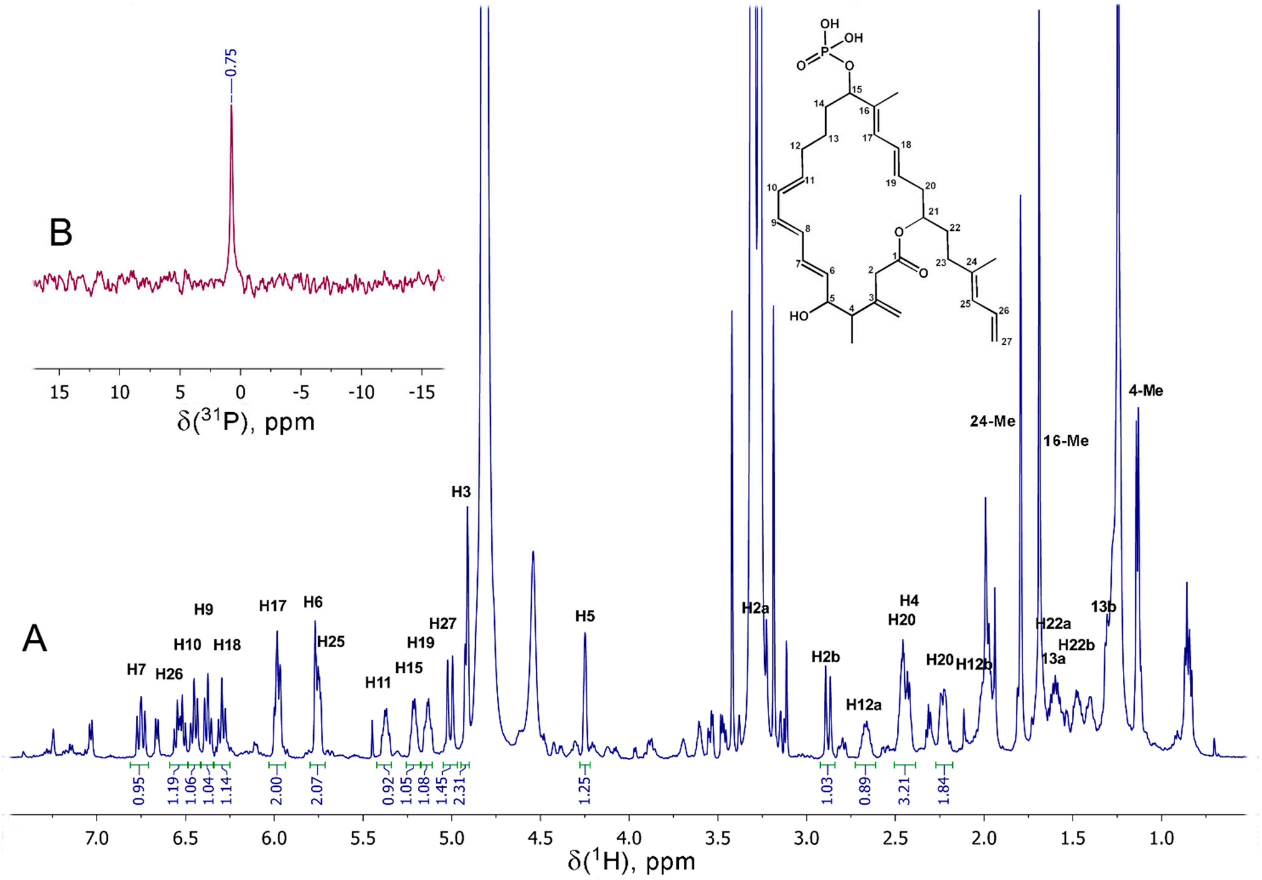

Figure S5.

NMR characterization of oxydifficidin in CD3OD at 298 K. (a) ^{1}H NMR spectrum with key resonances labeled. (b) ^{31}P NMR spectrum.

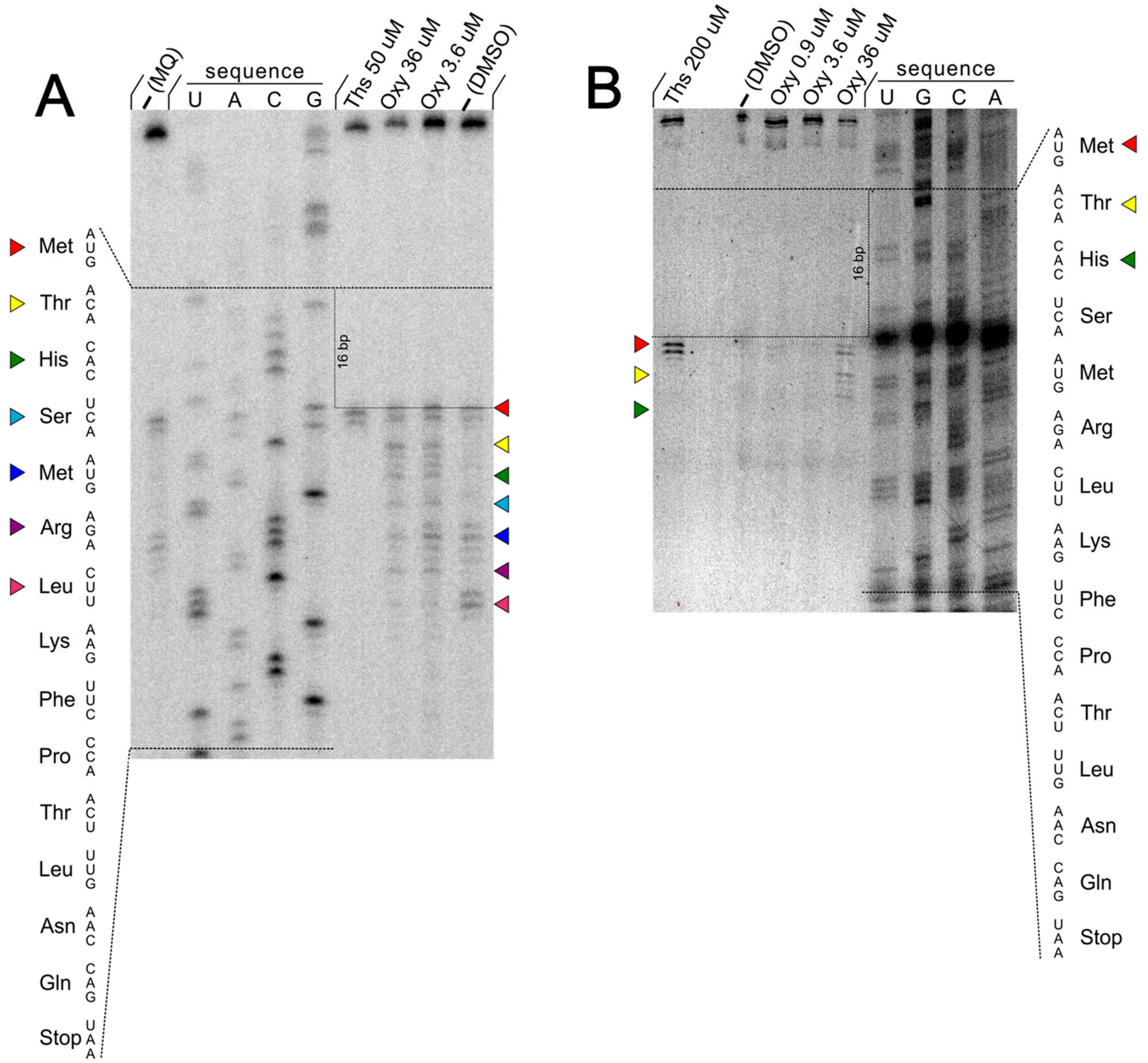

Figure S6.

Fluorescent and radioactively labeled toeprinting assays showing the effect of oxydifficidin on ribosome positioning on the ermDL mRNA template. (A) Toeprinting assay with radioactively labeled primer and (B) with fluorescently labeled primer of ErmDL template in the presence or absence (-) of Oxy at various concentrations and control antibiotic thiostrepton (Ths). Ths inhibits initiation (red arrow), while Oxy did not cause ribosome stalling at specific mRNA sites but instead caused non-specific pauses distributed across nearly every codon in the first part of the transcript.

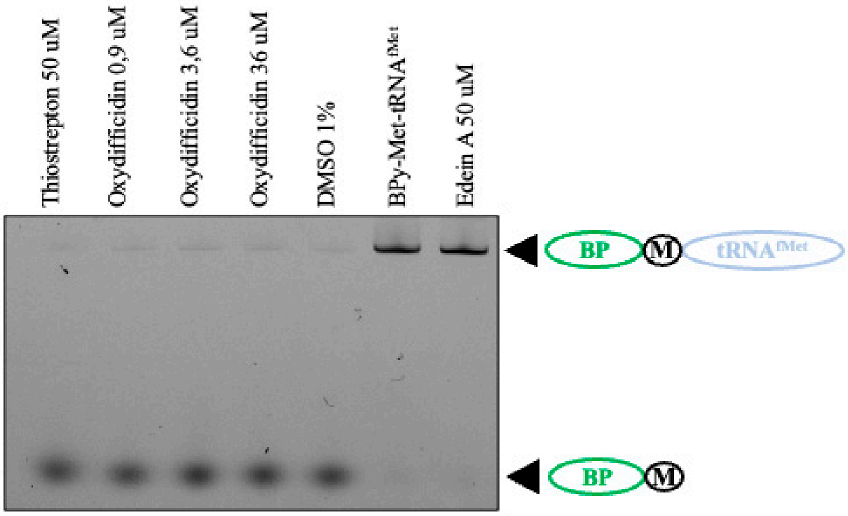

Figure S7.

Effect of oxydifficidin on translation initiation of Met-BODIPY-labeled peptide. An M-coding mRNA was translated in the presence of BPY-Met-tRNA^{fMet} by PURExpress *in vitro* translation Kit. Separation of translation products were performed in 10% UREA-PAGE. BPy-Met-tRNA^{fMet} itself, DMSO 1% - control without drugs, thiostrepton 50 uM, edein A 50 uM as positive control, oxydifficidin with 0.9, 3.6 and 36 uM final concentrations. BPY is marked with a green oval— BODIPY label; black circle—methionine; blue oval—tRNA^{fMet}.

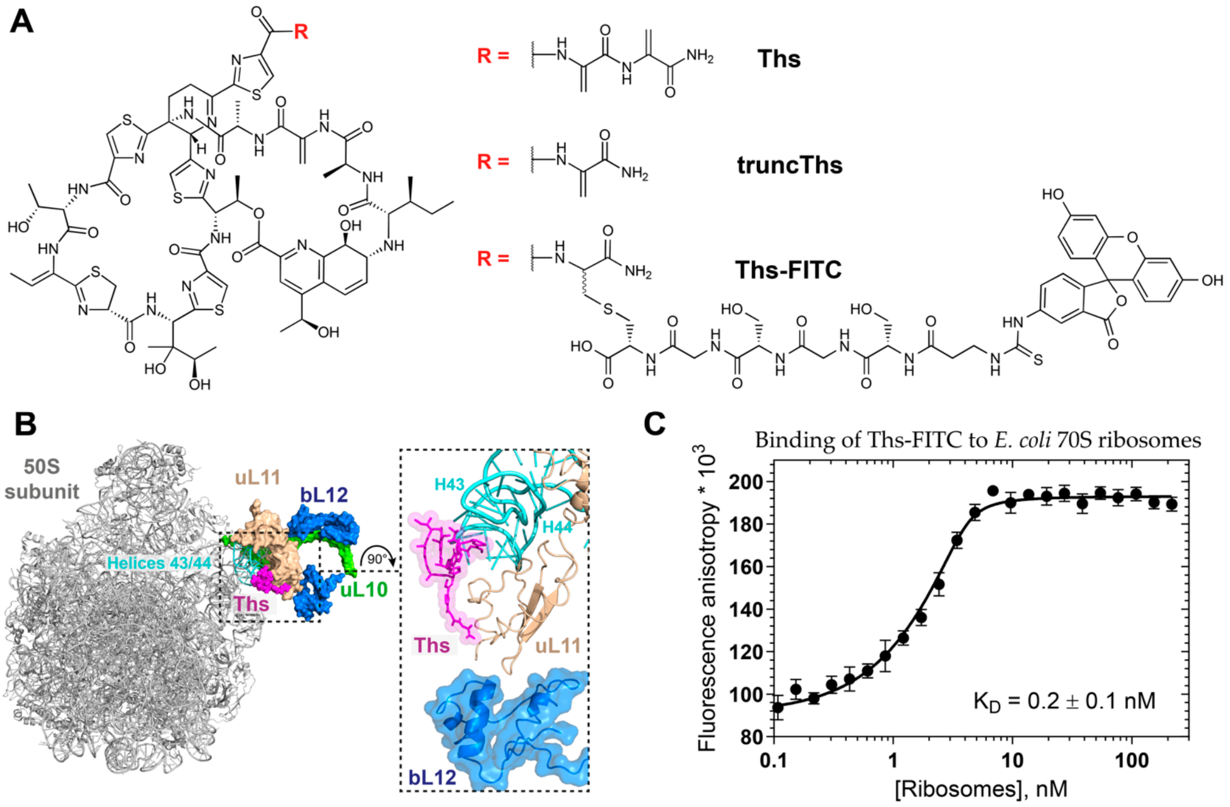

Figure S8.

Fluorescent thiostrepton derivative (Ths-FITC) and its binding to bacterial ribosomes. (**A**) Structures of thiostrepton (Ths), its truncated analogue (truncThs), and the fluorescent derivative (Ths-FITC). (**B**) Binding site of Ths (magenta) on the 50S ribosomal subunit of *Deinococcus radiodurans* (grey) (PDB ID: 3CF5). Ths interacts with ribosomal protein uL11 (beige) and 23S rRNA helices H43 and H44 (cyan), and is positioned near the stalk protein bL12 (blue). The location of bL12 was determined by superimposing the 50S subunit onto the structure of 70S *Thermus thermophilus* ribosome in complex with elongation factor G trapped in the post-translocational state (PDB ID: 4V5F). (**C**) Binding of Ths-FITC to *E. coli* 70S ribosomes measured by fluorescence anisotropy. Data represent means of four independent replicates; error bars indicate SD. The apparent dissociation constant (K_D) with CI (α = 0.05) is shown

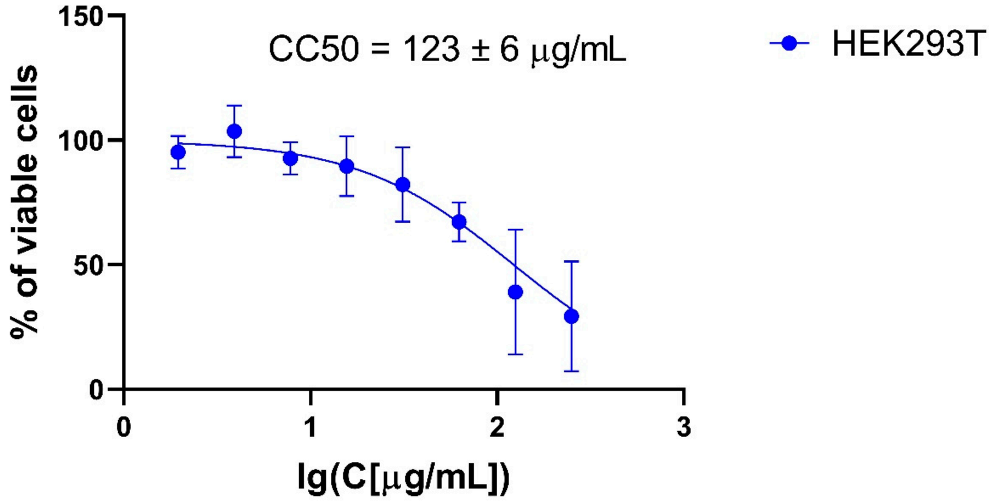

Figure S9.

MTT test results of oxydifficidin on HEK293T cell line viability. The results are exhibited with error bars representing standard deviation (SD). The SDs were counted between three replicas automatically in the process of dose-response curve fitting. Final SEM (standard error of the mean) of data was calculated by Graphpad Prism 8 to be approximately 4.1% of CC50, which is generally acceptable error (SEM is usually acceptable up to 0.15-0.2).

Table S1.

The activity of enzymes and the utilization of carbohydrates by *B. velezensis* strains

	EV17	*B. velezensis* FZB42[1]	*B. velezensis* NRRL B-41580[T 2]	K-3618
Acid production from carbohydrates				
L-Arabinose	—	—	—	—
Cellobiose	+	+	n/d	+
Galactose	—	+	n/d	—
Glucose	+	+	+	+
Inositol	—	—	+	w
Fructose	+	+	n/d	+
Lactose	+	+	+	+
Maltose	+	+	n/d	+
Mannitol	+	+	+	+
Mannose	+	+	+	+
Raffinose	+	+	n/d	+
Rhamnose	—	—	n/d	—
Sorbitol	+	+	+	+
Sucrose	+	+	+	+
Trehalose	+	+	n/d	+
Xylite	—	—	n/d	—
Xylose	w	—	n/d	w
Enzymes				
B-galactosidase	+	—	n/d	+
Phenylalanine deaminase	—	n/d	—	—
Arginine decarboxylase	w	n/d	—	w
Lysine decarboxylase	—	n/d	—	—
Ornithine decarboxylase	—	n/d	—	—
Urease	—	n/d	—	—
Indole production	—	—	—	—
Utilization of				
Malonate	+	n/d	n/d	+

[1] According to Borriss with collegues [https://doi.org/10.1099/ijs.0.023267-0], the carbohydrate utilization panel of FZB42 does not differ from those described for the type strain *Bacillus amyloliquefaciens* DSM 7 [https://bacdive.dsmz.de/strain/598]

[2] https://doi.org/10.1099/ijs.0.63310-0

Table S2.

Primers and templates. Overlapping areas of primers for pDualrep2.1 KanR obtaining are colored in yellow and green.

Name	Sequence
pdualrep2_fwd	AGCCTACACGCATCGATTATTGAAGCATTTATC
pdualrep2_rev	GTTGGGCTTACCAAGTTTACTCATATATACTTTAG
KanR_fwd	AAACTTGGTAAGCCCAACCTTTCATAGAAGG
KanR_rev	AATCGATGCGTGTAGGCTGGAGCTGCTTC
NV1	GTTATAATGAATTTTGCTTATTAAC
ermCL	ACTAATACGACTCACTATAGGGAGTTTTATAAGGA<br>GAAAAAATATGGGCATTTTTAGTATTTTTGTAATCA<br>CACAGTTCATTATCAACCAAACAAAAAATAATAAT<br>ATAAAAAAAGTGATAGAATTCTATCGTTAATAAGC<br>AAATTCATTATAACC
ermDL	ACTAATACGACTCACTATAGGGAGTTTTATAAGGA<br>GAAAAAATATGACACACTCAATGAGACTTAAGTTC<br>CAACTTTGAACCAGTAATAATAATAAAAAAAGTG<br>TAGAATTCTATCGTTAATAAGCAAAATTCATTATA<br>CCC

References

[1] M.V. Rodnina, W. Wintermeyer, GTP consumption of elongation factor Tu during translation of heteropolymeric mRNAs., Proc. Natl. Acad. Sci. U.S.A. 92 (1995) 1945–1949. https://doi.org/10.1073/pnas.92.6.1945.
